# Predictive modelling of heating and cooling degree hour indexes for residential buildings based on outdoor air temperature variability

**DOI:** 10.1038/s41598-023-44380-4

**Published:** 2023-10-13

**Authors:** Joanna Kajewska-Szkudlarek

**Affiliations:** Institute of Environmental Engineering, Wrocław University of Environmental and Life Sciences, Grunwaldzki Square 24, 50-363 Wrocław, Poland

**Keywords:** Engineering, Environmental sciences, Projection and prediction

## Abstract

Heating and cooling degree hours (HDH and CDH) are weather-based technical indexes designed to describe the need for energy requirements of buildings. Their calculation is the simplest method to estimate energy demand, providing the pattern of internal temperature variations in a building in response to weather conditions. The aim of the study is HDH and CDH prediction for Wrocław, Poland, based on outdoor air temperature using machine learning methods: artificial neural networks and support vector regression (ANN and SVR). The key issues raise in the study are: a detailed analysis of the most significant temperature lags (from 1 to 24 past hours) serving as predictors for modelling and an assessment of the impact of the database clustering on its accuracy. The best results are obtained with the clustering approach. The best predictor is the outdoor temperature observed 1 and 24 h before forecast demand (R^2^ = 0.981 and 0.904 for heating degree and cooling degree hours indices, respectively). Models with the highest quality are created using ANN, and the lowest with SVR. Prediction of heating/cooling degree hour indices provides building demand in advance, does not require knowledge about its characteristics, and expresses the possible impact of regional climate modifications.

## Introduction

Heating, ventilation, and air conditioning (HVAC) in buildings consume the main part of the energy produced^1–3^ and this will not change in the next decades^4^. In the face of global warming, winter in moderate climate is becoming less severe^3,5^, which affects the reduction of heating demand^6^; but summer is becoming hotter and requires a longer cooling period^5,7^. According to Eurostat^8^ in the EU the need for heating in 2021 was one tenth lower while the need for cooling was three times higher than in 1979. However, the building sector has a substantial importance in reducing energy consumption following the idea of an energy-efficient, low-emission and climate-friendly economy^9^. It is implemented by improving building insulation, using modern HVAC systems and, depending on the climate, heating or cooling demand prediction^10^. Forecasting the amount of heating and cooling demand leads to rational system’s operation^11,12^, which allows not only to reduce energy losses but also operational costs, and the volume of pollution as well as dust emission^13^ while maintaining thermal comfort. To estimate it, the most common method is to search for correlations between weather conditions and heating or cooling consumption^1,14^. The modern approach in that field consisted of using machine learning techniques^11,12^. Although various meteorological parameters are used as input for the modelling, the most important element in demand forecasting is the temperature of the outdoor air^15,16^.

Calculating the heating or cooling degree day (HDD, CDD) and heating or cooling degree hour (HDH, CDH) indices is the simplest method of estimating the energy demand of buildings^17^. They provide the pattern of internal temperature variation in response to weather conditions^18^, since they are the positive difference between the outdoor temperature and the indoor comfort temperature^3^. These indices are aggregated to days, months, years, and multi-year periods. It is a comprehensive, easy to calculate and apply method, does not require knowledge about the geometric and thermophysical characteristics of the building, and expresses the possible impact of regional climate modifications on energy demand^3^. For this reason, it is widely used in the energy industry.

Although prediction of heating/cooling demand is not a novel idea, prediction of HDH and CDH indexes has not yet been commonly discussed. The aim of the study was to fill this gap and to use machine learning methods to provide their prediction models based on hourly temperature time series from the moderate climate location of Wrocław, Poland, in the period 2010–2020. It is difficult to find such an extensive database in the literature on this subject, which initially covered 96 432 hourly air temperature values. Generally, heating and cooling load predictions are created based on the one heating or cooling season^1,12,15,19^.

Most of the forecasts of heating loads are based on various meteorological parameters and past loads; however, the present research aim to limit to minimum the number of predictors taking into account^20^ and^21^, which stated the need for simplicity in the model structure as the main principle for creating models with optimised parameters. Therefore, the created models are built only on the basis of the past hourly outdoor temperature as the most commonly measured meteorological parameter. The specific key issues raised in the study are detailed analysis of the most significant time lags in temperature (from 1 to 24 past hours) served as predictors for modelling and assessment of the impact of the database clustering into subsets with similar thermal conditions on modelling accuracy. Research results are also meant to indicate whether machine learning gives models with better accuracy than simple regression method.

## Material and methods

The scheme of the present research is as follows.

1. calculation of HDH and CDH indexes,
2. predictor selection (past temperature lags) for HDH and CDH indexes using linear regression (LR) modelling and the Pareto method,
3. creation of ANN and SVR (artificial neural networks and support vector regression) predictive models for degree hour (DH) indexes with various combinations of predictors,
4. splitting the database into subsets using cluster analysis (CA),
5. creation of ANN and SVR predictive models for HDH and CDH in clusters for selected predictors from 2).

Wrocław is located in the south-western part of Poland and its climate is described by characteristics typical of a transitional climate of mid-latitude zones, resulting in high climate variability. The city is the fourth largest in Poland and is subject to additional modifications, which are typical for large urban-industrial agglomerations^22^. Wrocław is located at 51.10°N and 16.88°E while its elevation is 121 m.

The initial research database covers hourly values of ambient air temperature from the period 1.01.2010 to 31.12.2020. It is provided by the Institute of Meteorology and Water Management (IMGW) of the Wrocław-Strachowice airport. According to WMO, station ID is 124,240.

The primary data set is a matrix of 96.432 lines containing air temperature in the actual hour and lagged temperatures from 1 to 24 passed hours (potential predictors – independent variables). Air temperature values in each hour are used to calculate hourly differences between comfort temperature and actual temperature and their series is a dependent, explained variable. They are the base for calculating HDH and CDH indexes for 11-year period 2010–2020 (modified formulas 1 and 2). It is respectively 66,113 and 5858 values and the corresponding lagged temperature values that served as predictors.

The obvious is that hourly air temperature time series will give highly accurate degree hour indexes prediction since these indices are calculated based on ambient temperature. However, the key issue raises in this study is to assess the most important time-lags in temperature (from 1 to 24 passed hours) that give prediction model with the best quality.

The calculation of the heating degree day and hour indexes includes the average room temperature and the base temperature, which is the lowest air temperature that does not require indoor heating. The base temperature value depends on several factors related to the building and the ambient environment. In the general climatological approach, this value is constant and equals 15.0 °C. According the Eurostat^23^, the HDD calculation formula is shown below. In the present research, based on^24^, heating degree hours are calculated using the same methodology.

1 $$ {\text{If}}\;{\text{ T}}_{{\text{m}}} \le {15}.0^\circ {\text{C }}\;{\text{Then }}\left[ {{\text{HDD }} = \, \Sigma_{{\text{i}}} \left( {{18}.0^\circ {\text{C }}{-}{\text{ T}}^{{\text{i}}}_{{\text{m}}} } \right)} \right] \, \;{\text{Else}}\; \, \left[ {{\text{HDD}} = 0} \right] \;{\text{Where }}\;{\text{T}}^{{\text{i}}}_{{\text{m}}} \; {\text{is }}\;{\text{the}}\;{\text{ mean}}\;{\text{ air }}\;{\text{temperature }}\;{\text{of}}\;{\text{ day }}\;{\text{i}}. $$

The cooling degree day and hour indexes calculation relies on average room temperature, and the base temperature, which is the highest air temperature that does not require indoor cooling. The base temperature value is constant and is set at 24.0 °C and the CDD is calculated as follows^23^. In the present study, according to^24^, cooling degree hours are calculated using the same methodology.

2 $$ {\text{If}} \;{\text{T}}_{{\text{m}}} \ge {24}.0^\circ {\text{C}}\;{\text{ Then}}\; \, \left[ {{\text{CDD }} = \Sigma_{{\text{i}}} \left( {{\text{T}}^{{\text{i}}}_{{{\text{m}} }} {-}{ 21}.0^\circ {\text{C}}} \right)} \right] \, \;{\text{else }}\;\left( {{\text{CDD }} = \, 0} \right) \;{\text{Where}}\;{\text{ T}}^{{\text{i}}}_{{\text{m}}} \;{\text{is }}\;{\text{the}}\;{\text{ mean}}\;{\text{ air}}\;{\text{ temperature}}\;{\text{ of}}\;{\text{ day}}\;{\text{ i}}. $$

The basis for the research is the multivariate linear regression modelling which is used to analyse the multivariate data. In this approach, the author assumes that the predicted HDH and CDH values are a linear combination of predictors (air temperature lags from 1 to 24 h). More specifically, linear combination of hourly differences between average indoor and actual temperature series, which serve for degree hour indexes calculation as well as temperature in past 1 to 24 h. A potential set of 24 predictors is presented on the Pareto charts, and those with *p* < 0.05 are considered significant.

To assess the impact of past thermal conditions on model quality, cluster analysis (k-means) is implemented. It creates groups of objects that are similar to each other in terms of a predefined measure. To determine the optimum number of clusters, a cost sequence analysis is executed for a tenfold cross-validation with a number of clusters between 2 and 25 and 5% minimum decrease. Clusters are determined on the basis of predictors (air temperature lags) that are set in the previous step. By increasing the number of clusters, the cost sequence decreases, which provides 5 clusters for HDH and 6 for CDH indices. Each cluster describes different past ambient temperature conditions that the most strongly influence the dependent variable.

For degree hour indices prediction, the two top machine learning techniques are implemented: multilayer perceptron (MLP), which is the type of artificial neural networks, and support vector regression, which belongs to support vector machines (SVM). The results obtained are compared with multivariate linear regression models.

To avoid overfitting, the data are divided into 70% training and 30% testing sets.

Models were created for:

1. all database and all past air temperature lags from 1 to 24 h as predictors,
2. all database and optimum set of predictors selected using the Pareto method (9 for HDH and 11 for CDH),
3. all database and 1 and 24 h temperature lags as predictors,
4. separate clusters similar in terms of past temperature conditions (5 for HDH and 6 for CDH) using an optimum set of predictors.

The artificial neural networks analyses employ learning process using a backpropagation algorithm, a Broyden-Fletcher-Goldfarb-Shanno (BFGS) technique, and weight reduction in the Weigend method. Various number of learning epochs (from 11 to 1312) and a varied number of hidden neurones (6–14) are implemented. From the set of models obtained, the one that gives the best accuracy is chosen.

Additionally, HDH and CDH indexes are predicted using SVR with the radial basis function (RBF) kernel. The parameters of the models created are ε = 0.1 and C = 10.0. The RBF is determined by γ (width of the kernel function), which, and the number of support vectors, are variable. The selection criterion for them is the maximum quality of the model.

The accuracy of the created models is assessed based on: mean squared error (MSE), mean absolute error (MAE), mean absolute percentage error (MAPE), and coefficient of determination (R^2^). In the case of MSE, MAE and MAPE the lower values mean the higher accuracy, for R^2^ the higher values, and close to 1, indicate a better fit of the model.

## Results

### Heating and cooling degree hour indexes for Wrocław, Poland

**Figure 1:**
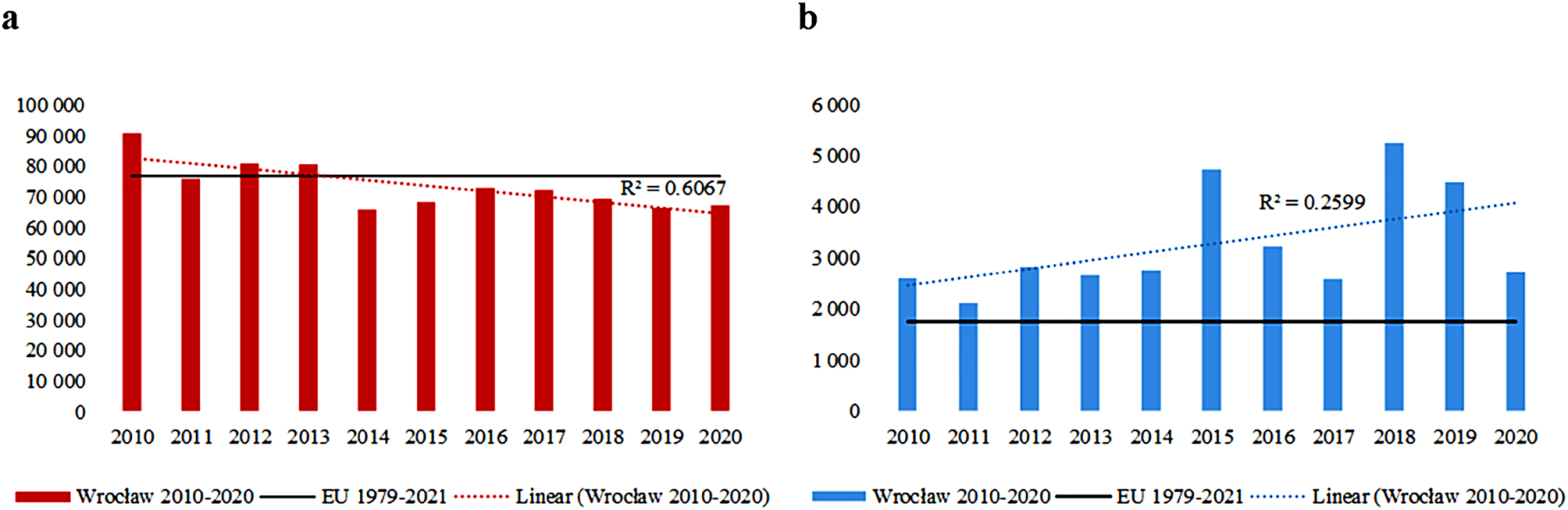
Annual HDH (**a**) and CDH (**b**) indexes for Wrocław in 2010–2020 and for EU in 1979–2021 period.

In Fig. 1 annual sums of HDH (red as need for heating) and CDH (blue as need for cooling) for Wrocław in the period 2010–2020 compared to the average value for the EU in 1979–2021 are presented. The yearly sums of heating degree hour indices show a significant downward trend (R^2^ = 0.6067) while cooling degree hours the slight upward trend (R^2^ = 0.2559). The highest values for HDH are noted in 2010 (almost 90,000) and in 2012 and 2013, and for this two years it is close to the EU mean multiyear sum (about 80,000).

For CDH, the highest annual sums are observed in 2015, 2018, 2019, and these were more than twice the mean annual sum for EU in the period 1979–2021 (1752).

### Predictor selection

**Figure 2:**
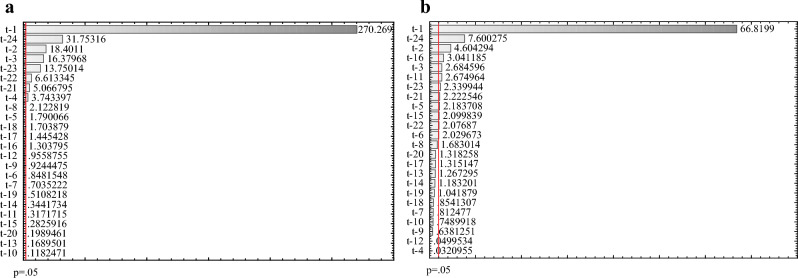
Pareto chart for (**a**) HDH (**b**) CDH.

The significance of the predictors for the degree hours’ forecasting is presented in the Pareto charts and air temperature lags with values greater than *p* = 0.05 are considered important (Fig. 2). The two most substantial, in both cases (a, b), are temperatures from 1 and 24 past hours with different combinations of other lags. For HDH the set of significant input variables consists of nine, while for CDH of eleven predictors for modelling. In general, the most important hourly lags are close to actual (1–6 h) and are related to the temperature the day before (21–24 h), which refers to the cyclical nature of daily changes in ambient air temperature.

### Database clustering

Splitting the whole database into clusters and building separate sub models for them is considered a method to improve the accuracy of the heating load predictions^25,26^ and in other applications^27,28^.

**Figure 3:**
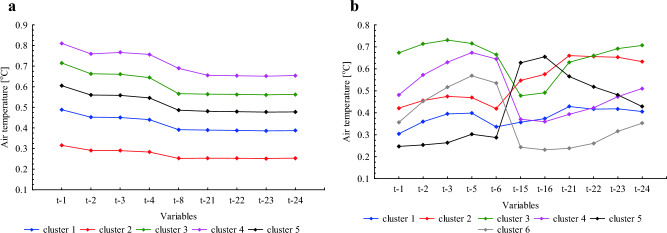
Normalized mean values of predictors in clusters for HDH (**a**) and CDH (**b**).

The creation of clusters for the HDH and CDH database is based on past hourly temperature lags selected using the Pareto method. Thus, each cluster represents different past ambient temperature conditions, which most strongly influence the dependent variable. The normalised mean values of the predictors in each group are presented in Fig. 3. The optimal number of clusters is 5 for HDH and 6 for CDH.

For HDH clusters are established in a simple way. Starting from cluster 2 (red line), each subsequent cluster includes cases with higher temperature, both near and far from the actual, while cluster 4 (pink line) concentrates the highest mean values of the predictors analysed. Cluster 5 (black line) groups the average temperature of all cases (Fig. 3a).

In the case of CDH, clustering based on air temperature is more complicated. The mean values of the predictors are presented in Fig. 3b. For example, group 5 clusters cases with the lowest temperature close to the actual temperature (from t-1 to t-6), the highest temperature in the middle of the previous day (t-15, t-16), and the average temperature almost the day before (from t-21 to t-24). Cluster 6 includes the average temperature from t-1 to t-6 and the lowest temperature in the later hours (from t-15 to t-24).

**Figure 4:**
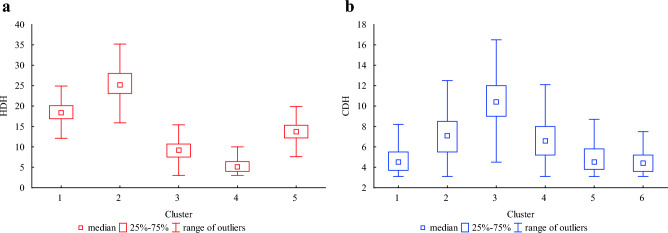
Box and whisker plots for HDH (**a**) and CDH (**b**) in clusters.

The explained variable distribution is different between clusters as it is significantly dependent on ambient air temperature conditions. The highest median for HDH and the largest dispersion are observed in the second cluster which is the one with the lowest temperature, while the lowest median and dispersion are observed in the fourth cluster, which is characterised by the least severe temperature conditions. The spread in clusters 1, 3, and 5 is similar; however, the median is the lowest in cluster 3 while the highest in group 1, which corresponds to the second most and the second least severe in terms of temperature cluster, respectively (Figs. 3a, 4a).

In the case of CDH, the largest range of outliers and median value are observed in the third group, where t-1 to t-6 are the highest and t-21 to t-24, while t-15 and t-16 were average. The smallest dispersion and median value are noticed in cluster 6, where the average thermal conditions are observed for the close past hours (from 1 to 6) and the most severe for the earlier hours (15–24) (Figs. 3b, 4b).

### Predictive modelling

The modelling results and quality of the created machine learning and LR models are evaluated based on accuracy metrics in the testing subsets that contain data that are not involved in the learning process. In Tables 1 and 2 these values, which cover the lowest MSE, MAE, MAPE, as well as the highest R^2^, are marked in grey.

**Table 1:** Modelling results for HDH and CDH.

	All database											
	HDH						CDH					
	LR		MLP		SVR		LR		MLP		SVR	
	Learn	Test	Learn	Test	Learn	Test	Learn	Test	Learn	Test	Learn	Test
MSE	0.686	0.777	0.587	0.676	0.841	0.944	0.572	0.690	0.461	0.610	0.563	0.709
MAE	0.573	0.623	0.530	0.581	0.684	0.731	0.536	0.561	0.478	0.515	0.538	0.579
MAPE	7.044	8.494	5.822	6.762	7.518	8.092	12.484	10.426	8.415	8.842	9.530	9.981
R^2^	0.982	0.973	0.985	0.977	0.978	0.968	0.906	0.883	0.924	0.897	0.909	0.881

	Selected predictors											
	HDH						CDH					
	LR		MLP		SVR		LR		MLP		SVR	
	learn	test	learn	test	learn	test	learn	test	learn	test	learn	test
MSE	0.687	0.779	0.608	0.687	0.865	0.961	0.573	0.691	0.466	0.607	0.610	0.726
MAE	0.574	0.623	0.540	0.588	0.702	0.747	0.536	0.562	0.478	0.512	0.572	0.594
MAPE	8.314	8.427	5.925	6.697	7.453	8.199	34.152	10.436	8.381	8.772	10.179	10.187
R^2^	0.982	0.973	0.984	0.976	0.978	0.968	0.906	0.883	0.923	0.898	0.901	0.879

	Clusters											
	HDH						CDH					
	LR		MLP		SVR		LR		MLP		SVR	
	Learn	Test	Learn	Test	Learn	Test	Learn	Test	Learn	Test	Learn	Test
MSE	0.676	0.720	0.589	0.645	1.136	1.242	0.547	0.627	0.483	0.555	0.494	0.596
MAE	0.572	0.600	0.533	0.564	0.807	0.845	0.521	0.535	0.484	0.508	0.495	0.517
MAPE	6.474	7.024	5.784	6.134	9.164	9.697	10.334	9.869	8.494	8.746	8.911	9.243
R^2^	0.982	0.978	0.984	0.981	0.970	0.963	0.911	0.891	0.922	0.904	0.920	0.897

	1 h and 24 h lags											
	HDH						CDH					
	LR		MLP		SVR		LR		MLP		SVR	
	Learn	Test	Learn	Test	Learn	Test	Learn	Test	Learn	Test	Learn	Test
MSE	0.982	1.121	0.919	1.046	2.479	2.417	1.192	1.320	1.045	1.183	1.476	1.581
MAE	0.690	0.750	0.667	0.724	1.306	1.275	0.838	0.881	0.768	0.812	0.999	1.034
MAPE	8.946	10.879	7.144	8.022	12.578	12.931	15.665	15.948	13.094	13.692	16.564	16.987
R^2^	0.974	0.961	0.976	0.964	0.970	0.958	0.804	0.777	0.828	0.800	0.807	0.779

**Table 2:** Modelling results for HDH and CDH in individual clusters.

	HDH cluster 1						CDH cluster 1					
	LR		MLP		SVR		LR		MLP		SVR	
	Learn	Test	Learn	Test	Learn	Test	Learn	Test	Learn	Test	Learn	Test
MSE	0.525	0.566	0.458	0.502	0.650	0.719	0.307	0.296	0.247	0.269	0.266	0.279
MAE	0.475	0.509	0.443	0.477	0.592	0.634	0.421	0.413	0.382	0.400	0.405	0.406
MAPE	2.564	2.783	2.399	2.619	3.211	3.497	9.578	8.854	8.258	8.445	8.794	8.553
R^2^	0.910	0.904	0.921	0.915	0.904	0.896	0.833	0.806	0.866	0.824	0.858	0.820

	HDH cluster 2						CDH cluster 2					
	LR		MLP		SVR		LR		MLP		SVR	
	Learn	Test	Learn	Test	Learn	Test	Learn	Test	Learn	Test	Learn	Test
MSE	0.611	0.559	0.537	0.535	0.658	0.608	0.452	0.527	0.365	0.485	0.442	0.529
MAE	0.513	0.503	0.482	0.487	0.567	0.549	0.486	0.532	0.447	0.511	0.488	0.520
MAPE	1.922	2.025	1.815	1.957	2.148	2.231	11.707	9.559	6.963	7.595	8.508	9.379
R^2^	0.961	0.936	0.966	0.939	0.959	0.930	0.903	0.868	0.922	0.878	0.906	0.869

	HDH cluster 3						CDH cluster 3					
	LR		MLP		SVR		LR		MLP		SVR	
	Learn	Test	Learn	Test	Learn	Test	Learn	Test	Learn	Test	Learn	Test
MSE	0.747	0.801	0.659	0.729	1.267	1.351	1.225	1.982	1.236	1.770	1.170	1.936
MAE	0.606	0.644	0.570	0.614	0.873	0.911	0.707	0.833	0.704	0.812	0.667	0.807
MAPE	7.033	7.467	6.640	7.102	9.924	10.347	7.305	10.011	7.169	8.519	6.797	8.900
R^2^	0.933	0.928	0.941	0.935	0.890	0.882	0.879	0.788	0.878	0.806	0.886	0.787

	HDH cluster 4						CDH cluster 4					
	LR		MLP		SVR		LR		MLP		SVR	
	Learn	Test	Learn	Test	Learn	Test	Learn	Test	Learn	Test	Learn	Test
MSE	0.716	0.813	0.588	0.702	1.277	1.465	0.730	0.760	0.681	0.668	0.646	0.704
MAE	0.625	0.667	0.562	0.612	0.865	0.919	0.618	0.617	0.585	0.576	0.578	0.604
MAPE	13.270	15.053	10.952	11.766	18.559	19.982	10.229	10.147	9.511	9.251	9.306	9.677
R^2^	0.758	0.742	0.801	0.777	0.605	0.578	0.811	0.795	0.824	0.819	0.833	0.812

	HDH cluster 5						CDH cluster 5					
	LR		MLP		SVR		LR		MLP		SVR	
	Learn	Test	Learn	Test	Learn	Test	Learn	Test	Learn	Test	learn	test
MSE	0.691	0.705	0.620	0.638	1.323	1.445	0.386	0.348	0.323	0.301	0.337	0.322
MAE	0.575	0.585	0.544	0.552	0.891	0.923	0.478	0.471	0.439	0.446	0.451	0.453
MAPE	4.296	4.338	4.080	4.101	6.683	6.904	10.696	10.110	9.271	9.334	9.703	9.557
R^2^	0.873	0.863	0.886	0.876	0.767	0.730	0.801	0.795	0.834	0.823	0.827	0.809

							CDH cluster 6					
							LR		MLP		SVR	
							Learn	Test	Learn	Test	Learn	Test
							0.404	0.350	0.295	0.285	0.336	0.304
							0.471	0.449	0.415	0.409	0.432	0.410
							11.670	10.855	9.199	9.307	9.638	9.372
							0.735	0.720	0.807	0.771	0.780	0.755

For the whole database and all of the predictors (temperature lags from 1 and 24 h), the R^2^ for the model equals 0.977 for HDH and 0.897 for CDH indexes. In both cases, the best quality is obtained with the neural approach; however, the linear and support vector regression results are only slightly worse. In the next step, the model is built for the predictors selected with the Pareto method. For CDH, the results are slightly better but for HDH slightly worse in the case of all fit metrics analysed, except for MAPE and HDH for which it amounts to 6.697 (previously it was 6.762).

For the overall model after clustering, the accuracy is better compared with the results described above for both degree hour indexes (R^2^ equals to 0.981 and 0.904, respectively).

The predictor selection analysis demonstrates that the most important are 1 and 24 h temperature lags, so at the present stage such predictors are taken into account to assess whether it is possible to maintain the accuracy of the models by reducing the number of input variables. However, the quality of the models created for such a combination shows the worst quality of all that have been analysed so far, which is 0.964 and 0.800 (R^2^) for HDH and CDH (Table 1).

The accuracy of the predictive models created in particular clusters indicates that the results are different depending on the accuracy metrics taken into account. The best quality for HDH models in terms of MSE (0.502) and MAE (0.477) is obtained in cluster 1 (the one with the second lowest temperature); however, according to MAPE (1.957) and R^2^ (0.939) it is cluster 2 (the one with the lowest temperature). Furthermore, the worst model accuracy, evaluated by MSE (0.729) and MAE (0.614), is observed in cluster 3 (the one with the second highest temperature), but based on MAPE (11.766) and R^2^ (0.777) in cluster 4 (the one with the highest temperature) (Table 2).

In the case of CDH, the smallest MSE and MAE values (0.269 and 0.400 respectively) are obtained in cluster 1 and the largest in cluster 3 (MSE = 1.770; MAE = 0.807) while the lowest MAPE (7.595) and highest R^2^ (0.878) are obtained in cluster 2. The worst model quality assessed based on MAPE (9.334) is observed in cluster 5, and R^2^ (0.771) in cluster 6.

Almost in all cases, predictive models with the highest quality, both in learning and testing subsets, are created by artificial neural networks, multilayer perceptron. SVR gives the best accuracy (in the learning subset) only for clusters 3 and 4 in the CDH modelling, whereas the LR results are a little worse in all cases. As in the models created for the whole database, better results are obtained in HDH predictions compared to CDH (Table 2).

## Discussion

To the best of the author’s knowledge, the present research is the first attempt to predict degree hour indexes. However, the results are compared with other heating/cooling load forecasting models in the literature created using machine learning methods.

Zhao and Liu^11^ who combined the wavelet transform, SVM, and PLS to predict the load in an office building obtained consistent results with those of this study for single techniques (the accuracy of the best HDH model was 6.13 and for CDH 8.75%). Depending on the prediction horizon, the MAPE values ranged from 6.13 to 9.40 for cooling and from 5.79 to 13.42% for heating load. However, when they used hybrid models (Wavelet-PLS-SVM), the quality was 2.60–9.87 and 3.99–12.19, respectively. Slightly higher accuracy resulted from taking historical load as one of input variables, which is not practised in this research. According to R^2^, their predictions were as accurate as presented in this study (0.8 to 0.9). From meteorological parameters, they considered relative air humidity, air temperature, and solar radiation, but temperature lags included only previously 1–3 h.

Ding et al.^15^ also studied the influence of exterior condition on heating load (outdoor air temperature and humidity, solar radiation, and wind speed) from the predicted time to the past 24 h. Using correlation analysis in the preliminary stage of forecasting, they concluded that the air temperature during the past 1 to 8 h is the factor that affects the heating load. The MLP and SVR were used in predictive modelling, which provides a convergent conclusion as in this research, that neural networks outperform SVR. Their forecasts were accurate at about 66 – 94% (R^2^) and the results indicated that the use of meteorological parameters that describe external weather conditions significantly improves the quality of the models created.

Referring to the use of weather data in prediction, Wei et al.^19^ formulated the same conclusion. They used seven machine learning algorithms to predict the heating load in Shanghai, China based on data from electrical power, thermal, and meteorological sensors, as well as from weather forecasts. The Analysis showed that SVR gives the best performance described by MAPE of 5.21%. That was similar to the present results, but the DH forecasts were the most accurate using MLP and the SVR provided the least. Furthermore, the authors claimed that increasing historical data sets does not improve the performance of the models and recommended its length to be 28 h, while here 24 h is applied, reflecting the cyclical nature of daily changes in ambient air temperature.

Ling et al.^1^ who employed historical thermal, humidity, solar and heating load conditions for office building heating load prediction using BPNN and SVR did not consider air temperature and humidity influence on heating load separately, but comprehensively as the temperature-humidity index (THI). However, the THI indices of 1 to 24 lags were analysed with non-hourly correlation analysis and as the most influenced THI-1, THI-2, and THI-24 were selected. Their approach provided BPNN models with significantly lower accuracy (45–65% and SVR with 45–80%) compared to the present results.

Eguizabal, Garay-Martinez and Flores-Abascal^29^ proposed the ARX model to provide a one hour ahead prediction of the heating load for Bilbao and Madrid considering lagged values of ambient temperature, solar irradiation, and heat load. After an analysis of 1–12 h lags, the authors concluded that the most important are past conditions from 1–4 h. They did not study conditions for 24 h, which proves to be important in the present research. However, the precision of their models described with R^2^ values was in the range of 0.92 to 0.94, which was only slightly worse.

Lim and Kim^30^ used an extensive data set of input meteorological variables, covering, among others, outdoor air temperature, to predict the cooling load in office buildings in Seoul, South Korea. However, they analysed only 1 and 2 h lags to create forecasts 1–5 h in advance using multiple regression models. The performance of the models evaluated based on R^2^ was 0.6–0.7 depending on the size of the building; the larger the building, the higher the quality of the model.

Dahl et al.^31^ who created heating load models for Aarhus, Denmark taking into account lagged load, meteorological parameters, calendar, as well as holidays data using OLS, MLP, and SVR concluded that the most important ambient temperature lag is 4 h. The best performance was obtained with SVR combining weather, calendar, and holiday data, which was MAPE = 6.4%.

The clustering of the database used in this study improves the accuracy of the prediction models. Its magnitude depended on the type of quality metrics. When comparing the modelling results in the testing subset (Table 1) for the whole database created based on selected predictors (selected predictors) with those after clustering and the same predictors (clusters), the quality increases for HDH by approximately 6.1, 4.2, 8.4, and 1.0% (for MSE, MAE, MAPE, and R^2^, respectively). For CDH, the observed improvement is 8.6 for MSE and for other measures about 1.0%. According to heating load forecasting, Lu et al.^25^ who implemented clustering based on temperature and person behavior obtained a significantly higher increase of 51.2% using segmented modelling while Yuan et al.^32^ who clustered data based on ambient air thermal conditions obtained an improvement of 41.2 and 56.6%. In the case of the predictions of cooling load for Guangzhou, China, Chen et al.^33^ proved the increase of 34.6% in MAPE after data segmentation.

The present research results in that field do not demonstrate such an impressive improvement in the accuracy of the models after clustering; however, it is a consequence of the fact that the preliminary results were already of high quality (R^2^ = 0.9).

## Conclusions

Prediction of heating and cooling demand using machine learning techniques is a way to optimise the operation of HVAC systems to meet the needs of building occupants and maintain thermal comfort. Calculation of degree hour indexes is an intuitive and comprehensive method of loading estimation, providing the pattern of internal temperature variations in a building in response to exposure to the weather conditions, and it is widely used in the energy industry.

The present research concerns the prediction of HDH and CDH indices based on the hourly ambient air temperature for Wrocław, Poland. The strength of the created models is that they do not require a priori knowledge on the building, large computational effort as well as wide input datasets, only air temperature measurement, and they can be used to forecast any heat load independently of climate conditions.

The higher accuracy in HDH modelling is achieved in the test set for the overall clusters (MSE = 0.645, MAE = 0.564, MAPE = 6.134, R^2^ = 0.981) based on nine air temperature lags, among which the most important are t-1 and t-24.

The same most significant predictors are taken into account in CDH forecasting; however, the whole selected set amounted to 11 input variables. Both degree hour indexes models with the highest quality are created using neural networks, and the lower with SVR. The goodness of fit measures for the best CDH model (after clustering) are 0.555, 0.508, 8.746, and 0.904 (MSE, MAE, MAPE, R^2^, respectively).

Furthermore, reducing the number of temperature lags in the predictors set to the most important t-1 and t-24 provides significantly lower precision. Although LR models have a worse fit to real data than MLP, all three methods (either SVR) show similar efficiency.

The k-means clustering procedures emerge 5 and 6 clusters (HDH and CDH, respectively) with different past thermal conditions that affected the performance in the created groups. In general, for HDH the highest values of accuracy metrics are obtained in clusters with the lowest ambient temperature, as well as the lowest in groups with the highest temperature. In case of CDH, the performance of the model depends on thermal conditions in close lags (from t-1 to t-6) and in later hours (from t-15 to t-24) that clearly influenced the clustering process.

The estimations of heating demand in Poland are more important than cooling due to climate conditions, as the main part of the energy sold to households is used to heat buildings during the winter period (stat.gov.pl). However, as a result of contemporary climate change and heat waves in summer that occur more and more frequently, the demand for energy in ventilation and air conditioning systems is also increasing. Therefore, both the predictions of HDH and CDH indexes can be successfully used to estimate the demand for heating and cooling throughout the year. The proposed models might be only applied in residential and office buildings, as when other building applications are involved, internal gain and occupancy can play a significant source of error.

The aim of the study is to create heating and cooling demand prediction models based only on past outdoor air temperature as the main meteorological parameter that influence heating/cooling load. However, in the future research, it is planned to be implemented other important parameters’ (relative air humidity, wind speed and sunshine duration) analysis.

## Data Availability

The datasets used and analyzed during the current study are available from the corresponding author upon reasonable request.
